# Ergonomics in handheld and robot-assisted camera control: a randomized controlled trial

**DOI:** 10.1007/s00464-019-06678-1

**Published:** 2019-02-11

**Authors:** Paul J. M. Wijsman, Lennert Molenaar, Cas D. P. van‘t Hullenaar, Bas S. T. van Vugt, Wim A. Bleeker, Werner A. Draaisma, Ivo A. M. J. Broeders

**Affiliations:** 1 Department of Surgery, Meander Medical Center, Maatweg 3, Amersfoort, The Netherlands; 2 Technical Medicine, University of Twente, Enschede, The Netherlands; 3 Department of Surgery, Wilhelmina Hospital Assen, Assen, The Netherlands; 4 Department of Surgery, Jeroen Bosch Hospital, ‘s Hertogenbosch, The Netherlands; 5 Robotics and Mechatronics, University of Twente, Enschede, The Netherlands

**Keywords:** Laparoscopic camera holder, Ergonomics, Active robotic camera steering, AutoLap™ system

## Abstract

**Background:**

Laparoscopic surgery potentially increases the physical burden to operating theater personnel and can cause physical discomfort. This study aims to evaluate if a robotic camera holder (AutoLap™ system) can improve ergonomics for the surgeon and the camera assistant during laparoscopic procedures.

**Methods:**

A total of thirty cases were included and randomized (15 AutoLap™, 15 control). Five types of surgery were included: right hemicolectomy, fundoplication, sigmoid resection, rectopexy, and low anterior resection. The posture of the surgeon and assistant was photographed during predefined steps of the procedure. MATLAB was used to calculate angles relevant for the RULA score. The RULA score is a validated method to evaluate body posture, force and repetition of the upper extremities. Two investigators assessed the RULA score independently. Three subjective questionnaires (SMEQ, NASA TLX, and LED) were used to assess mental and physical discomfort.

**Results:**

No differences in patient characteristics were observed. Sixteen fundoplications, seven right hemicolectomies, five sigmoid resections, one rectopexy, and one low anterior resection were included. The mean RULA score of the surgeon was comparable in both groups, 2.58 (AutoLap™) versus 2.72 (control). The mean RULA score of the assistant was significantly different in both groups, with 2.55 (AutoLap™) versus 3.70 (control) (*p* = 0.001). The inter-observer variability (ICC) was excellent with 0.93 (surgeon) and 0.97 (assistant). The questionnaires showed a significant difference in physical discomfort for the assistant. The LED and SMEQ score were significantly lower in the robotic group. The NASA TLX demonstrated a significant reduction in scores in all domains when using robotics with the exception of the mental domain.

**Conclusion:**

Use of the AutoLap™ system shows improvement in ergonomics and posture of the first assistant, and ergonomics of the surgeon are not affected. Furthermore, the subjective work load is significantly reduced by using a robotic camera holder.

**Trial registration number:**

NCT0339960, https://clinicaltrials.gov/ct2/show/study/NCT03339960?term=autolap&rank=5.

## Background

Laparoscopic surgery can lead to an increased physical burden for operating room personnel. Several studies demonstrate that physical discomfort is frequently reported by all members of the laparoscopic surgical team [1–5]. Surgeons, as well as their first assistant who steer the laparoscope, often work in unfavorable positions. The ergonomics of the first assistant are frequently compromised while displaying an optimal image for the surgeon. This is usually caused by standing outside the central working axis causing torsion in the back and asymmetrical burden to legs and shoulders. Recently, robotic camera holders were introduced into the field of minimal invasive surgery. This can potentially lead to improved ergonomics [6].

In this study, the AutoLap™ system (Medical Surgery Technologies Ltd., Yokneam, Israel) was deployed. A detailed description of this robotic camera holder has been previously described [7]. By using an active robotic camera holder, the surgeon is able to steer the camera. The first assistant is relieved of the task of camera control and can focus on other tasks such as tissue traction. Moreover, the ergonomic posture of the first assistant can also be improved as camera control is an ergonomically unfavorable task. Because the ergonomics of the operating theater and especially the first assistant are frequently overlooked, it is of major importance to measure and improve ergonomic scores. Therefore, the purpose of this study is to investigate the role of a robotic camera holder in relation to the ergonomics of the surgeon and first assistant.

## Methods

In this prospective multicenter randomized controlled trial, ergonomics and posture of surgeons and their first assistants were analyzed. Measurements were executed in two different hospitals, the Meander Medical Center, Amersfoort and Wilhelmina Hospital, Assen. The local ethics committee (Medical Research Ethics Committees United) and board of directors of both hospitals approved the study and no informed consent was required. Three surgeons and multiple first assistants participated in this trial.

Preliminary data gathered in the Meander Medical Center have demonstrated an improvement in RULA score when using the robotic system when compared to standard laparoscopy (RULA score of 4). A difference of 10% in RULA score (0.7 RULA score difference) was thought to be clinically relevant and a sample size calculation (power 80%, significance level 5%) revealed a sample size of 30 cases, assuming equal variance.

In total, thirty cases were included and randomized by using stratified block randomization (15 AutoLap™, 15 control). Random block sizes were determined by Castor EDC software. Five different procedures were included: laparoscopic right hemicolectomy, laparoscopic fundoplication, laparoscopic sigmoid resection, laparoscopic rectopexy, and laparoscopic low anterior resection. These procedures were selected due to the fact that they are a good reflection of the daily practice of an endoscopic surgeon. Secondly, these five operations are regularly performed with the robotic camera holder in both hospitals. All three surgeons have extensive experience with the robotic system and have completed their learning curve.

Sagittal and dorsal postures of the surgeon and the first assistant were recorded by taking photographs of predefined steps in every procedure. The position of the operating theater personnel and the screen position were standardized. The surgeons and first assistant were aware that their posture was recorded during the surgery; however, to ensure the natural posture was captured, they were not pre-warned of the exact time pictures were to be taken and were not given any additional instructions intra-operatively.

**Fig. 1:**
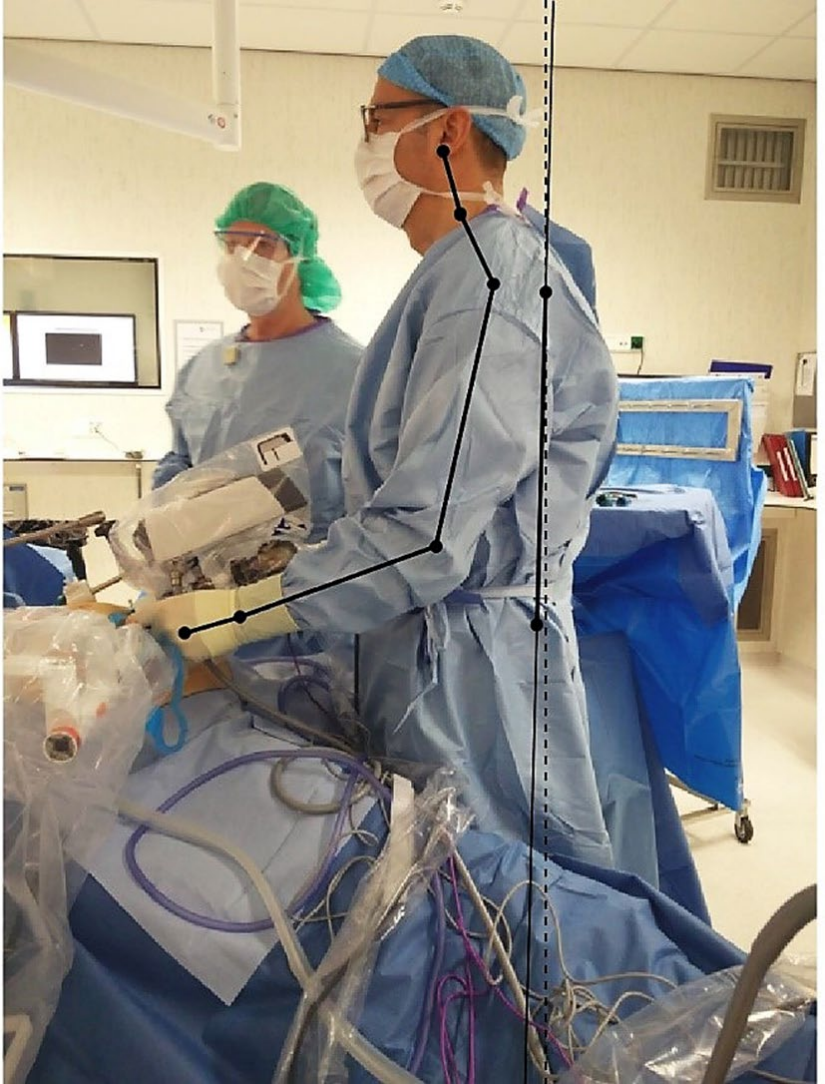
Measured angles with the MATLAB script during a laparoscopic fundoplication

All photographs were analyzed using Matlab 2017b (The MathWorks, Nattick, MA, USA). Software scripts were customized and incorporated in this study. Using these software scripts, the angles of the shoulder, elbow, wrist, neck, and trunk were calculated (Fig. 1). This was performed at each predefined step in every single procedure. All five types of operations were divided in seven different tasks to assure that a similar task and posture was captured in every operation. The postures were captured at the start of every step. An example of the steps during hemicolectomy right are as follows: (1) mobilization of the caecum, (2) dissection of Toldt’s line, (3) making the subcolic tunnel, (4) dissection of the ileocolic artery, (5) dissection of the gastrocolic ligament, (6) mobilization of the hepatic flexure, and (7) completion of the anastomosis.

Additionally, all joint angles were converted to the Rapid Upper Limb Assessment score (RULA, “Appendix” 01, Ergoplus, Indianapolis, USA) [8]. Because laparoscopic surgery generally leads to unilateral strain, the arm with the highest RULA score was incorporated for analysis. The RULA score defines four types of outcome: 1–2 (acceptable posture), 3–4 (further investigation, change may be needed), 5–6 (further investigation, change soon), and 7 (investigate and implement change). Two investigators calculated and assessed all RULA scores independently. Subjective validated questionnaires (Subjective Mental Effort Questionnaire (SMEQ) [9], NASA TLX [10], and Local Experienced Discomfort (LED) [11]) were also completed by all participants to assess mental and physical discomfort.

The data were analyzed using SPSS (IBM SPSS Statistics for Windows, Version 24.0, Armonk, NY). Independent samples t test was used to analyze statistical significance. Outliers, distribution, and variance were checked to assure the assumptions associated with this test were not violated. Outliers were visually checked with a boxplot. To determine if the data are normally distributed, a Q–Q plot was drafted and the Shapiro–Wilk test for normality was executed. Lastly, homogeneity of variance was assumed with the Levene’s test of equality of variances.

## Results

A total of 30 cases were included, 15 in the AutoLap™ group and 15 in the control group. Three surgeons, all with extensive laparoscopic experience and well trained with the AutoLap™ system, performed all surgical procedures. 17 cases were performed in the Meander Medical and 13 cases were performed in the Wilhelmina Hospital. No differences in age and BMI were observed. Mean age was 60.1 (± 13.4) and mean BMI 26.6 (± 4.1). The demographic data of the patients are displayed in Table 1. Five types of surgery were included: 16 fundoplications (seven AutoLap™ cases), seven right hemicolectomies (four AutoLap™ cases), five sigmoid resections (two AutoLap™ cases), one rectopexy, and one low anterior resection (both AutoLap™ cases).

**Table 1:** Demographic data of the patients

	AutoLap	Control	Total	*p* Value
Age	60.7	59.6	60.1	0.83
BMI	27.5	25.6	26.6	0.21
Procedures				
Fundoplication	7	9	16 (53%)	
Right hemicolectomy	4	3	7 (23%)	
Sigmoid resection	2	3	5 (17%)	
Low anterior resection	1	0	1 (3%)	
Rectopexy	1	0	1 (3%)	

The mean RULA score of the surgeon was equal in both groups, 2.58 (± 0.47) in the AutoLap™ group and 2.72 (± 0.53) in the control group, *p* = 0.45. However, the mean RULA score of the first assistant was significantly lower in the AutoLap™ group, 2.55 (± 0.54) versus 3.69 (± 0.57), *p* = 0.001 (shown in Table 2). The intraclass correlation coefficient between the two investigators was 0.93 for the surgeon RULA score and 0.97 for the assistant RULA score.

**Table 2:** Mean RULA scores for the surgeon and assistant

RULA—scores	Mean	*p* Value	Standard deviation	*N*
Surgeon				
Robotic	2.58	0.45	0.47	15
Control	2.72		0.53	15
Assistant				
Robotic	2.55	0.001	0.54	15
Control	3.69		0.57	15

Subjective questionnaires, including the LED, SMEQ, and NASA TLX, displayed identical scores for the surgeon in both groups. The mean LED score (post-surgery minus pre-surgery) for the surgeon was 1.1 (± 2.30) versus 1.6 (± 2.0), and mean SMEQ score was 49.7 (± 20.3) versus 43.2 (± 19.1) (AutoLap™ vs. control, respectively). The raw NASA TLX was equivalent on all domains (mental, physical, temporal, performance, effort, and frustration) in both groups.

For the first assistant, the subjective workload was significantly lower in the AutoLap™ group for all three questionnaires. The mean LED score (post-minus pre-operative) was 0.73 (± 3.65) versus 10.5 (± 13.2), *p* = 0.010, the mean SMEQ score 28.9 (± 23.3) versus 53.3 (± 23.5), *p* = 0.008, in favor of the AutoLap™ group (see Table 3). The NASA TLX score was significant lower in the Autolap™ group on all domains except the mental domain. The mean mental score was 5.0 (± 2.9) versus 7.2 (± 3.9), *p* = 0.088, physical 4.1 (± 3.5) versus 9.0 (± 4.5), *p* = 0.002, temporal 2.8 (± 1.8) versus 5.5 (± 2.6), *p* = 0.003, performance 4.1 (± 2.2) versus 6.5 (± 3.1), *p* = 0.018, effort 4.9 (± 3.7) versus 8.1 (± 3.7), *p* = 0.024, and frustration 2.8 (± 1.8) versus 4.5 (± 2.8), *p* = 0.048. The NASA TLX scores are displayed in Table 4.

**Table 3:** Pre-operative, post-operative, and total LED scores and SMEQ scores for surgeons and assistant

Questionnaires	SMEQ		LED						*N*
			Pre		Post		Total		
	*M*	Std	*M*	Std	*M*	Std	*M*	Std	
Surgeon									
Robotic	49.67	20.31	0.57	0.90	1.70	2.80	1.13	2.29	15
Control	43.17	19.07	0.90	1.58	2.53	2.59	1.63	2.00	15
*p* Value	0.37						0.53		
Assistant									
Robotic	28.83	23.32	4.07	9.06	4.80	11.83	0.73	3.65	15
Control	53.33	23.45	2.20	4.04	12.67	12.97	10.47	13.18	15
*p* Value	0.008						0.010		

*Pre* pre-operative, *post* post-operative, *total* post–pre, *M* mean, *Std* standard deviation

**Table 4:** The raw NASA TLX with the six domains displayed

Q	NASA TLX												*N*
	Mental		Physical		Temporal		Performance		Effort		Frustration		
	*M*	Std	*M*	Std	*M*	Std	*M*	Std	*M*	Std	*M*	Std	
Surgeon													
Robotic	7.70	4.09	6.23	3.41	4.63	2.57	3.30	1.58	7.90	4.74	4.57	3.71	15
Control	6.57	3.23	5.83	2.37	3.93	1.22	3.33	0.90	7.07	3.12	4.63	3.10	15
*p* Value	0.407		0.712		0.348		0.944		0.574		0.958		
Assistant													
Robotic	4.97	2.86	4.10	3.46	2.83	1.78	4.07	2.24	4.93	3.67	2.77	1.79	15
Control	7.17	3.88	9.03	4.52	5.47	2.62	6.53	3.08	8.13	3.70	4.53	2.79	15
*p* Value	0.088		0.002		0.003		0.018		0.024		0.048		

## Discussion

The results of this study demonstrate that the ergonomics of the first assistant dramatically improves when using a mechanical camera holder such as the Autolap™ system. The disproportional effort of an assistant holding the laparoscopic camera in an unfavorable position is no longer required when a robotic camera holder is installed. Standing in a twisted or stooped position for a longer period, while keeping one hand perfectly still, can be avoided this way. The role of the first assistant alters; now they can concentrate on tissue traction or presentation of the surgical target area without worrying about steering and focusing the camera. This also enables the possibility to perform the operation as “solo surgery” (perform the operation with only two members: surgeon and assistant). Because the first assistant does not have to hold the laparoscope, the contribution to the operation is smaller. Besides, one hand is freed which makes it easier to also perform the role of scrub nurse and hand the instruments to the surgeon.

The ergonomic situation for the surgeon is not altered when using the Autolap™ system. Image quality however, a very important factor in laparoscopic surgery, may significantly improve. By using a camera holder, the captured image is continuously stable and in full focus, with a high and consistent quality. Furthermore, the surgeon is now exclusively responsible for steering the camera, being no longer dependent on the skills and qualifications of the first assistant. This was not part of our study, but in the study performed by Kavoussi et al. there were less inadvertent camera movements and rotations in the robotic group with the use of the AESOP (Computer Motion system) [12]. A study performed by Proske et al. also reported favorable image quality and stability when using a robotic camera holder [13]. Holländer et al. reported in a large study of 1033 procedures that eight out of nine surgeons preferred robotic to human assistance, mainly because of a steady image and self-control of the camera [14].

Of course, some drawbacks were reported when using a robotic camera holder. The large dimensions of the system can cause interference with laparoscopic instruments. Mostly, this can be reduced by placing the trocar ports a least 8–10 cm apart from each other. We would recommend future versions of the device should take this into account and we advocate reduction in the size of the camera holding device. Furthermore, by using a camera holder system which makes use of a parallelogram, the reach of the camera is limited. The range of motion is often limited to a maximum of 120° horizontally and 110° vertically. For most tasks during laparoscopic procedures, this range is not a limiting factor. However, when for instance mobilizing the splenic flexure when performing a laparoscopic left colectomy, readjustment of the camera holder is needed during the procedure. This might cause interference of the procedure, which can lead to distress, hindrance, or even a longer operation time. However, surgeons that are well trained and experienced with the system will easily overcome these issues.

As already mentioned in the methods, we included five very different laparoscopic procedures. These operations all have a different setup, trocar placement, operation steps, operative times, and to a lesser extent ergonomics. This might have caused heterogeneity bias in a small sample size of 30. However, a sub-analysis per procedure revealed no major discrepancies between the five procedures. The highest mean overall (robotic and laparoscopic combined) RULA score for the surgeon was during a rectopexy (3.42) and the lowest during a fundoplication (2.34). For the assistant, the highest mean overall RULA score was measured during a fundoplication (3.49) and the lowest during a rectopexy (2.0). These differences can be explained by the setup of the operation and the working axis. Standing outside the central working axis causes torsion of the back and asymmetrical burden of the legs and shoulders.

In this study, RULA scores were calculated to assess the ergonomic situation of the surgical team members. To calculate the RULA score, several variabilities should be considered. An important note to address is the natural posture of a person. When one tends to lean forward with the head during a procedure, this will be scored as ergonomically suboptimal. Besides, certain designated key steps of every surgical procedure were assigned and photographed. The operation theater personnel were not influenced in any way to capture their natural position. The calculation of the RULA score was performed afterwards. All photographs were shot by a single investigator to assure a standardized method of capturing the posture during each procedure. The postures of the team members were not always easy to identify on every single photograph. Moreover, the angle at which the photograph is taken is extremely important. If the photo is slightly out of the sagittal or dorsal plain, angle calculations can be difficult. Therefore, a second posture analysis was performed by an independent researcher. The intra-observer variability was close to 1.0, meaning a perfectly corresponding RULA score by both researchers.

For several reasons, only one arm was used for analysis of the RULA score. Firstly in most cases, only one arm is heavily burdened during surgery. In the control cases, this was always the arm that was holding the endoscope. Also, the LED questionnaire displayed increased scores in body parts at the side of the arm that was holding the scope. Secondly, in order to not falsely reduce the RULA scores, the mean of two arms was not used.

A qualitative approach would be highly informative regarding the reasons why the differences in the assistant group were seen. However, looking at the three subjective questionnaires (SMEQ, LED, and NASA TLX) we believe that many first assistants find it difficult to perform more than one task at the same time. When the first assistant is not in charge of controlling the camera, the SMEQ score is significant lower. This effect is also seen in the NASA TLX subdomain mental and temporal performance, effort, and frustration. Moreover, many assistants find camera control physical demanding. This effect is seen in the LED score and the NASA TLX physical subdomain.

When calculating RULA scores, one also should realize that maintaining a standing position for a longer period (mainly static posture) will increase the RULA score. Therefore, working in an operating theater automatically leads to higher workload scores. If this is combined with an ergonomic unfavorable position, scores tend to escalade. Therefore, ongoing attention and improvements for the high physical workload at the operation theater is of major importance. It will be one of the key elements to keep the surgical workforce ‘fit to perform’ over the next decades [15].

## Conclusion

The use of a robotic camera holder leads to a significant improvement in ergonomics for the first assistant. Moreover, the subjective work load is reduced by using an active robotic camera holder such as the AutoLap™ system. Ergonomics and work load of the surgeon are not affected.

## Figures and Tables

**Fig. 2:**
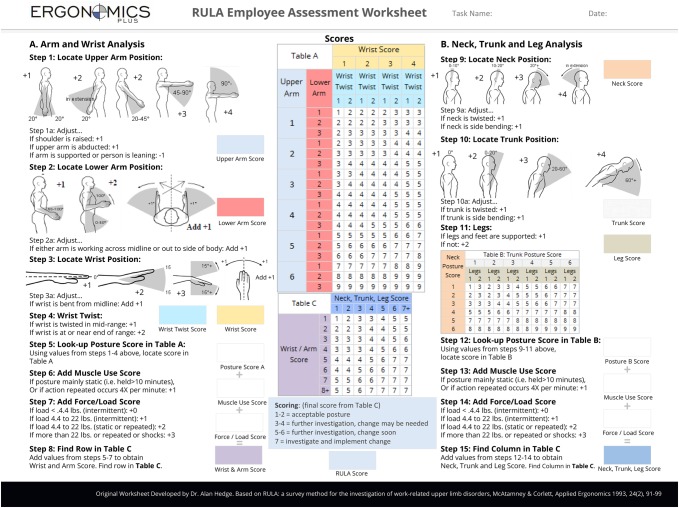
Rapid Upper Limb Assessment score worksheet used to assess the RULA score

## Appendix

See Fig. 2.
